# Brain Atrophy and Cognitive Impairment in Primary and Secondary Progressive Multiple Sclerosis Cohort—Similar Progressive MS Phenotype

**DOI:** 10.3390/ijms26178523

**Published:** 2025-09-02

**Authors:** Bartosz Gajewski, Małgorzata Siger, Iwona Karlińska, Igor A. Bednarski, Mariola Świderek-Matysiak, Mariusz Stasiołek

**Affiliations:** Department of Neurology, Medical University of Lodz, Kopcinskiego 22, 90-153 Lodz, Poland; bartosz.gajewski@stud.umed.lodz.pl (B.G.); iwona.karlinska@umed.lodz.pl (I.K.); igor.bednarski@umed.lodz.pl (I.A.B.); mariola.swiderek-matysiak@umed.lodz.pl (M.Ś.-M.); mariusz.stasiolek@umed.lodz.pl (M.S.)

**Keywords:** progressive multiple sclerosis, brain atrophy, cognitive impairment, progression, putamen, SDMT

## Abstract

The diagnosis and monitoring of progressive multiple sclerosis (PMS) require further development of fast and effective clinical tools. Relations between MRI-based brain atrophy measures and cognitive impairment in people with primary progressive and secondary progressive MS (PwPPMS, n = 20 and PwSPMS, n = 19, respectively) were investigated in a prospective study with follow-up after a mean 14.97 ± 4.67 months. MRI analysis showed that at baseline and follow-up in PwSPMS, the left thalamic fraction and corpus callosum fraction were significantly lower than in PwPPMS (baseline: 0.39 ± 0.04 vs. 0.44 ± 0.06, *p* = 0.0203 and 0.26 ± 0.05 vs. 0.30 ± 0.05, *p* = 0.0097; respectively and follow-up: 0.40 ± 0.04 vs. 0.44 ± 0.07, *p* = 0.0443 and 0.25 ± 0.06 vs. 0.30 ± 0.05, *p* = 0.0103, respectively). In contrast, only at baseline, PwPPMS had a significantly lower cerebellar white matter fraction (CWMF) than PwSPMS (1.83 ± 0.20 vs. 2.01 ± 0.24, *p* = 0.0132). No other significant differences were observed in the MRI fractions at either study time point or in the changes of the MRI fractions between the PwPPMS and PwSPMS. However, a significant decline in the right putaminal fraction was found during observation in PwSPMS (0.332% ± 0.05% vs. 0.328% ± 0.05%, *p* = 0.0479). Cognitive test scores and their changes did not differ significantly between the subgroups. Declines in the Brief Visuospatial Memory Test Revised in the whole PMS group (18.74 ± 7.43 vs. 17.03 ± 7.61, *p* = 0.0209) and in PwPPMS (19.50 ± 8.29 vs. 17.20 ± 7.72, *p* = 0.0338), as well as in the Brief International Cognitive Assessment for Multiple Sclerosis in PwPPMS (1.05 ± 0.89 vs. 1.25 ± 1.02, *p* = 0.0421), were observed. In both PwPMS and PwPPMS, a worsening on the Symbol Digit Modalities Test (SDMT) was associated with the reduction of fractions of white matter, cerebellum and right thalamus. SDMT performance also correlated with both gray matter fraction (GMF) and CWMF in the whole group, and with cerebellar gray matter fraction (CGMF) in PwPPMS. In PwSPMS, only Stroop Color and Word Test scores correlated with GMF and CGMF. In conclusion, subtle differences between PwPPMS and PwSPMS were detected both in MRI and neuropsychological parameters. Thus, our results indicate the need for a multicomponent attempt in characterizing progression in different clinical courses of MS.

## 1. Introduction

Multiple sclerosis (MS) is a chronic autoimmune disease of the central nervous system (CNS) involving demyelination, axonal damage and neurodegeneration [1]. According to the currently accepted criteria, the majority of people with MS (PwMS) are initially diagnosed with relapsing-remitting MS (RRMS) [2]. However, in 18.1% to 90.0% of people with RRMS (PwRRMS), the clinical course eventually changes to secondary progressive MS (SPMS), with median time from the first symptoms to conversion of 32.4 years [3,4,5,6,7]. Approximately 10% to 15% of PwMS are diagnosed with primary progressive MS (PPMS) [2]. People with PPMS (PwPPMS) and those with SPMS (PwSPMS) are referred to as people with progressive MS (PwPMS) [2]. Despite significant differences in the clinical course, a growing body of evidence indicates that MS is one disease, with progressive pathological processes undergoing continuously and beginning from the preclinical stages of the disease [8,9,10,11,12,13]. However, the knowledge regarding potential differences in progression characteristics between different clinical subforms of clinically progressive disease remains unsatisfactory.

Magnetic resonance imaging (MRI) constitutes a well-established paraclinical modality in the diagnostic workup of MS, as well as in the evaluation of therapeutic response and disease progression [14]. Although MRI characteristics in PwRRMS are very well described, data concerning this aspect in PwPMS is insufficient. The most distinctive feature of MRI in PwPMS is the brain and spinal cord atrophy [15]. Brain atrophy is not a uniform process—cortical gray matter (GM), deep GM and cerebellum are the most affected brain regions in PwPMS [16,17,18]. Also, longitudinal observations showed that annualized rate of cortical and deep GM atrophy is faster in PwPMS than in PwRRMS and control groups [16,17,18].

From an imaging perspective, a few differences between PwPPMS and PwSPMS have been published [15]. PwPPMS tend to have a low number of brain T2-lesions [15,19] as well as a low number of small-sized gadolinium-enhancing T1-lesions [20,21]. Also, the specific location of lesions—juxtacortical and cortical—has been shown to be more characteristic for PwPPMS than PwSPMS [19]. Studies focused on MRI diffusion reported a lower mean diffusivity in certain brain areas of PwPPMS than PwSPMS [22,23]. Moreover, diffused spinal cord abnormalities are more likely to be found in PwPPMS than PwSPMS [24]. PwSPMS, however, have more brain T1-hypointense lesions (black holes) than PwPPMS [25]. From a pathological point of view, PMS is characterized by a predominance of neurodegenerative over inflammatory changes [26]. In clinical practice, the most common approach to the evaluation of neurodegeneration is MRI-based atrophy assessment [27]. Although brain atrophy in PwPMS is a typical MRI feature, specific investigations of the differences in distribution of MRI-based brain atrophy between PwPPMS and PwSPMS are lacking [28,29]. Using voxel-based morphometry (VBM), Ceccarelli et al. [28] showed that PwSPMS had significantly lower GM volume in the thalamus (bilaterally), anterior lobe of cerebellum (bilaterally), superior and inferior colliculi and also left cuneus, left postcentral gyrus and middle occipital gyrus.

Despite a growing interest in PMS, differentiation of its subtypes based on a single MRI parameter or even a combination of parameters seems to be very difficult [15]. Therefore, in the evaluation of PwPMS, other markers such as the assessment of cognitive functions should be taken into consideration.

Cognitive impairment (CI) is a crucial clinical factor of MS [30,31]. It affects as many as 65% of PwMS [32] and can develop from the very beginning of the disease [33,34]. Notably, CI has a profound impact on the quality of life of PwMS [35] and is also a predictor of physical disability progression [36]. Cognitive decline of PwMS is observed predominantly in the domains of information processing speed (IPS), memory, attention and executive functions [37]. PwPMS are at a greater risk of CI [38,39] than PwRRMS, which could be attributed to more advanced neurodegeneration [37,40]. Five cognitive phenotypes of PwMS have been proposed: (1) preserved cognition, (2) mild–verbal memory/semantic fluency, (3) mild–multidomain, (4) severe–executive/attention, and (5) severe–multidomain, with particular clinical and MRI characteristics [41].

Associations between MRI and CI parameters have been evaluated in many studies [30,42,43,44,45,46,47,48]. They showed that cognitive decline has been associated with a number of MRI parameters, including T2- and T1-lesion number and volume, in both white matter (WM) and GM, as well as with the location of lesions (mainly in frontal, parietal and temporal lobes or cerebellum) [41,49,50]. Moreover, results from several investigations proved that brain atrophy (both global and regional) is strongly associated with CI in PwMS [42,47]. From a genetic standpoint, the impact of apolipoprotein E genotypes on brain atrophy and cognition in PwMS cannot be excluded [51,52,53,54]. However, most of these studies evaluated the correlations between brain atrophy and CI in PwRRMS, while data on these relationships in PwPMS remain more limited [55].

In our previous study (Gajewski B. et al. [56]), we did not identify any serum biomarkers specifically distinguishing PwPPMS from PwSPMS. Additionally, no correlation was found between the levels of serum biomarkers and the scores of neuropsychological tests in the investigated cohort of PwPMS. Taking this into consideration, currently we have extended our research and tested whether a combination of multiple regional brain atrophy MRI measures and cognitive parameters may better characterize disease progression in PMS subtypes and support the monitoring of patients.

## 2. Results

### 2.1. Study Group Characteristics

Fifty participants were included in the study. The measured parameters were assessed in two consecutive time points: (1) at baseline and (2) after follow-up of a mean 14.97 ± 4.67 months (median 14.42, IQR 13.0–16.75). Thirty-nine (78%) PwPMS (twenty PwPPMS and nineteen PwSPMS) completed all the follow-up examinations and were qualified for the statistical analysis. Four patients withdrew from the study during the follow-up and seven patients completed the follow-up procedures only partially due to personal reasons (three patients were followed up radiologically but not psychologically while four patients were followed up psychologically but not radiologically).

The baseline demographic and clinical characteristics of the study group are presented in Table 1. PwPPMS and PwSPMS did not differ significantly in age, sex or education level. PwSPMS had a significantly longer disease duration (20.05 ± 8.26 years vs. 8.00 ± 6.08 years, *p* = 0.00001) and a significantly higher EDSS score (5.63 ± 0.96 vs. 4.75 ± 1.16, *p* = 0.0137) than PwPPMS.

The majority of the participants (67%) received DMT, which was stratified as moderate-efficacy (MET; interferon beta, glatiramer acetate, dimethyl fumarate, teriflunomide) or high-efficacy therapy (HET; ocrelizumab, natalizumab) [57,58]; some patients were treated with other immunosuppressive agents (cyclophosphamide) or remained untreated (Table 1).

At follow-up, a significant increase in EDSS score was noted in the whole study group compared to the baseline assessment (5.18 ± 1.14 vs. 5.51 ± 1.16, *p* = 0.0343). Similarly to the baseline results, PwSPMS had a significantly higher EDSS score than PwPPMS at follow-up (5.92 ± 0.87 vs. 5.13 ± 1.28, *p* = 0.0287; Table 1).

### 2.2. MRI Results

At baseline, PwSPMS were characterized by a significantly lower LTF and CCF than PwPPMS (0.39 ± 0.04 vs. 0.44 ± 0.06, *p* = 0.0203 and 0.26 ± 0.05 vs. 0.30 ± 0.05, *p* = 0.0097, respectively), whereas CWMF was significantly higher in PwSPMS (1.83 ± 0.20 vs. 2.01 ± 0.24, *p* = 0.0132; Table S1).

Similarly to at baseline, at follow-up, LTF and CCF were significantly lower in PwSPMS than in PwPPMS (0.40 ± 0.04 vs. 0.44 ± 0.07, *p* = 0.0443 and 0.25 ± 0.06 vs. 0.30 ± 0.05, *p* = 0.0103, respectively; Table S1).

No other significant differences in the MRI fractions at both study time points or in the changes of the MRI fractions between PwPPMS and PwSPMS were observed (Table S1 and Table 2).

During the observation, a significant decline in RPF was found in PwSPMS (0.332% ± 0.05% vs. 0.328% ± 0.05%, *p* = 0.0479; Table S1). No other significant changes in the fractions of the assessed brain structures were noted (Table S1).

The statistical models identified disease duration as a significant predictor of brain atrophy only in the case of CCF and CWMF at follow-up in PwPMS (for details, see Tables S3 and S4 in the Supplementary Materials).

### 2.3. Analysis of Neuropsychological Tests

Based on BICAMS battery, thirty-one PwPMS (79%) were cognitively impaired at both study time points. We did not detect significant differences in the scores of the applied neuropsychological tests between PwPPMS and PwSPMS at either study time point (Table 3).

There were no significant differences in the changes of the individual tests between PwPPMS and PwSPMS during the observation (Table 4).

A significant decrease in the mean score of BVMT-R in the whole study group was shown (18.74 ± 7.43 vs. 17.03 ± 7.61, *p* = 0.0209; Table 3). Such decrease was also observed in PwPPMS (19.50 ± 8.29 vs. 17.20 ± 7.72, *p* = 0.0338; Table 3) but not in PwSPMS. Furthermore, a significant worsening on BICAMS battery was found in PwPPMS throughout the observation period (1.05 ± 0.89 vs. 1.25 ± 1.02, *p* = 0.0421; Table 3). The scores of the other neuropsychological tests did not significantly change during the study in either of the subgroups (Table 3).

### 2.4. Correlations Between the Changes of Neuroradiological and Neuropsychological Parameters

**Figure 1:**
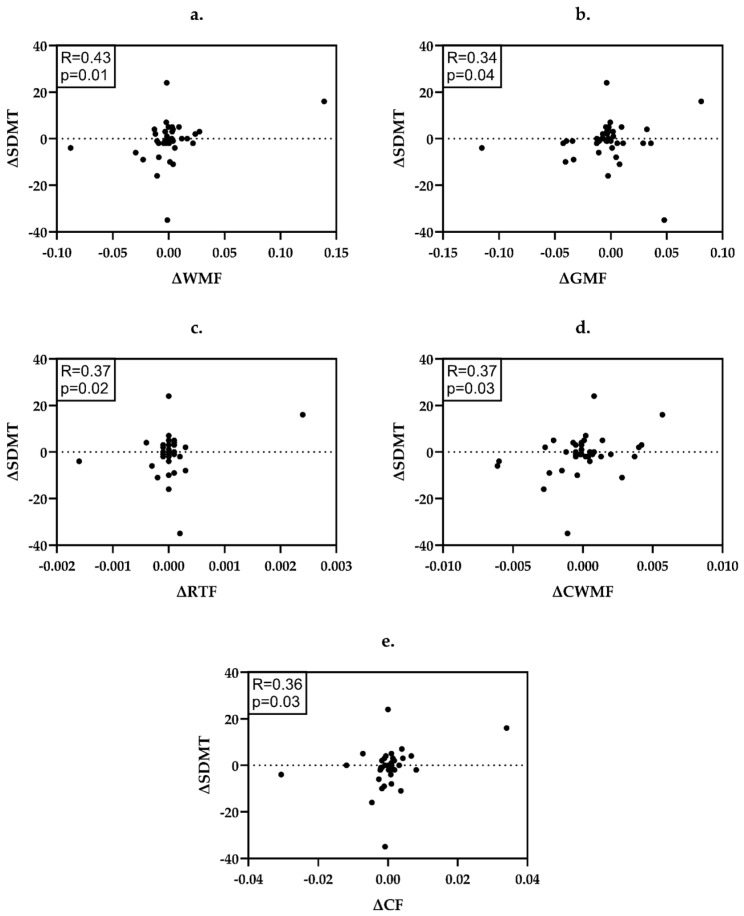
Correlations between the changes (∆) of neuroradiological and neuropsychological parameters in the whole study group. (**a**) ΔWMF and ΔSDMT; (**b**) ΔGMF and ΔSDMT; (**c**) ΔRTF and ΔSDMT; (**d**) ΔCWMF and ΔSDMT; (**e**) ΔCF and ΔSDMT. WMF—white matter fraction, GMF—gray matter fraction, RTF—right thalamic fraction, CWMF—cerebellar white matter fraction, CF—total cerebellar fraction, SDMT—Symbol Digit Modalities Test.

In the whole study group (Figure 1), the decrease of WMF (R = 0.43, *p* = 0.01), GMF (R = 0.34, *p* = 0.04), RTF (R = 0.37, *p* = 0.02), CWMF (R = 0.37, *p* = 0.03) and CF (R = 0.36, *p* = 0.03) was associated with the worsening in SDMT.

**Figure 2:**
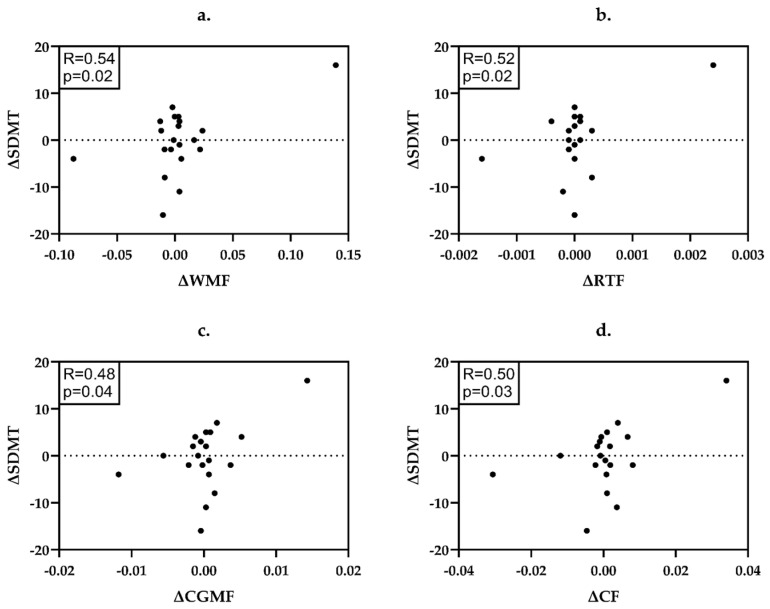
Correlations between the changes (∆) of neuroradiological and neuropsychological parameters in PwPPMS. (**a**) ΔWMF and ΔSDMT; (**b**) ΔRTF and ΔSDMT; (**c**) ΔCGMF and ΔSDMT; (**d**) ΔCF and ΔSDMT. PwPPMS—people with primary progressive multiple sclerosis, WMF—white matter fraction, RTF—right thalamic fraction, CGMF—cerebellar gray matter fraction, CF—total cerebellar fraction, SDMT—Symbol Digit Modalities Test.

In PwPPMS (Figure 2), the reduction of WMF (R = 0.54, *p* = 0.02), RTF (R = 0.52, *p* = 0.02), CGMF (R = 0.48, *p* = 0.04) and CF (R = 0.50, *p* = 0.03) was related to the worsening on SDMT.

**Figure 3:**
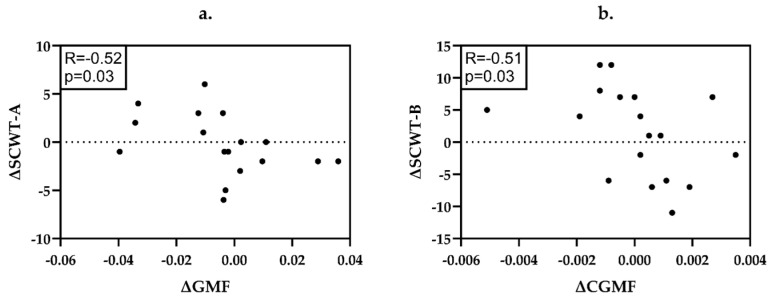
Correlations between the changes (∆) of neuroradiological and neuropsychological parameters in PwSPMS. (**a**) ΔGMF and ΔSCWT-A; (**b**) ΔCGMF and ΔSCWT-B. PwSPMS—people with secondary progressive multiple sclerosis, GMF—gray matter fraction, CGMF—cerebellar gray matter fraction, SCWT-A—Stroop Color and Word Test subtest A, SCWT-B—Stroop Color and Word Test subtest B.

In PwSPMS (Figure 3), the decrease of GMF was associated with the worsening on SCWT-A (R = −0.52, *p* = 0.03) while the decline in CGMF correlated with the deterioration on SCWT-B (R = −0.51, *p* = 0.03).

Detailed data on the statistical power and significance of the applied correlations may be found in Table S2 and Figure 1, Figure 2 and Figure 3.

## 3. Discussion

Effective assessment and monitoring of progression in PwMS requires multiple complementary methods. No single marker with high sensitivity and specificity, broadly accepted for this purpose, currently exists, therefore a multicomponent approach, including serum and cerebrospinal fluid (CSF) biomarkers, neuroimaging and neuropsychological evaluation might be useful. In our previous study, we did not find correlations between the molecular parameters or the results of neuropsychological tests and physical disability in PwPMS [56]. In the current research, we explored the application of other markers, namely a combination of MRI-based brain atrophy assessment and CI profiles in the monitoring of PMS progression, with possible distinction between PwPPMS and PwSPMS.

The present study demonstrated some differences in the volumetric parameters of specific brain regions between PwPPMS and PwSPMS as well as a different pattern of correlation between brain atrophy and cognitive parameters in these two subtypes of MS.

### 3.1. Brain Atrophy on MRI in PwPPMS and PwSPMS

Our study revealed that PwSPMS were characterized by a more pronounced atrophy of LTF than PwPPMS, at baseline and at the end of the study. The thalamus, a predominantly GM structure, is a crucial brain structure for sensation, movement, consciousness and cognition [59]. Thalamic atrophy was very extensively examined in PwMS [16,60,61,62]. It was detected already in the early stages of the disease, with continuous volume loss throughout its course [63]. Thalamic atrophy was named a ‘barometer’ of early neurodegeneration and total network disruption [64] in all MS subtypes [65], even in patients with radiologically isolated syndrome (RIS) [66]. Thalamic atrophy was associated with a range of clinical parameters and CI, and it is also accepted as a very sensitive marker of the disease progression [67]. Accordingly, it has been also established as a core endpoint in many MS clinical trials [68]. Our finding of lower LTF in PwSPMS than in PwPPMS stays in line with the results of the study held by MAGNIMS group [16], which encompassed 3604 MRI scans from 1214 PwMS and showed that among different MS phenotypes, the lowest deep GM volume (including thalamus) was found in PwSPMS. Our outcomes are also consistent with the data presented by Tobyne et al. reporting more advanced thalamic atrophy in PwSPMS than PwPPMS [69]. However, this observation is not fully consistent, as another research project showed no difference in the thalamus atrophy rate between PwPPMS and PwSPMS [70]. The difference in LTF between PMS subtypes can be explained in several ways. We may speculate that lower LTF in PwSPMS is a consequence of their longer disease duration. Many studies have shown that local thalamic atrophy is more severe in patients with longer disease duration [60,61,71]. However, in our study, disease duration did not influence the thalamic fractions. Differences in retrograde and anterograde neurodegeneration through the tracts connecting deep GM structures and cortical GM areas may contribute to lower LTF in PwSPMS than in PwPPMS [16]. Additionally, we cannot exclude the confounding effect of other factors; e.g., apolipoprotein E genotype [54].

We also found that CCF was lower in PwSPMS than in PwPPMS at both study time points. Corpus callosum (CC) is a WM structure and the largest of the brain’s commissures connecting both hemispheres [72]. It plays a pivotal role in conveying and integrating the signals of motor, sensory and cognition processes [72]. CC is a typical location for demyelinating MS lesions [73] while its atrophy is present from the very beginning of the disease, described even in clinically isolated syndrome (CIS) [74]. In PwMS, atrophy of CC is closely associated with the level of disability [75] and implicates a wide array of cognitive deficits in domains such as IPS, memory and executive functions [76]. CC atrophy is also an accepted predictor of cognitive and neurological progression in PwMS [76,77,78]. Furthermore, results from several studies indicated that CC atrophy in PwMS can also serve as a sensitive and specific marker to monitor the processes of neurodegeneration [77,79,80]. Similarly to our finding of lower CCF in PwSPMS than in PwPPMS, another study showed that the CC area was significantly lower in PwSPMS than in PwPPMS [29]. Lower CCF in our cohort of PwSPMS, as compared to PwPPMS, may be associated with longer disease duration. In contrast to thalamus measurements, a weak association of disease duration and CCF was presented in our study, although only at follow-up and in the whole study group (Table S4). Correlation between CC atrophy and disease duration was detected in several studies [75,81,82,83]. Also, differences of CC volumes between MS subtypes may be ascribable to the varying arrangement of CC thin fibers specifically prone to the atrophy processes, as was shown in a predominantly SPMS subgroup [84]. Additionally, distinct CC atrophy patterns might also result from the difference in the severity of Wallerian degeneration caused by pathology in the adjacent WM [85,86,87,88,89] or from pathology in GM, as a secondary effect [90].

On the other hand, we noted lower CWMF in PwPPMS than PwSPMS. The cerebellum is a hub for muscle tone, coordination and precision movement in CNS [91]. It also plays one of the key roles in cognition, and deficits of working memory, verbal fluency, attention or executive functions as results of cerebellar damage have been reported in PwMS [92]. Cerebellar atrophy was identified as a predictor of both physical and cognitive disease severity and disability, including IPS decline [93]. Data regarding differences of MRI-based cerebellum atrophy between PwPPMS and PwSPMS is very limited. In a study by Ceccarelli et al., atrophy of the cerebellum was more advanced in PwSPMS than in PwPPMS [28]. In another study, Cocozza et al. showed no differences in cerebellar atrophy between PwPPMS and PwSPMS [94]. As opposed to studies mentioned above, we detected lower CWMF in PwPPMS than in PwSPMS. The apparent differences may be associated with particular study populations but also result from a different methodology of atrophy assessment (e.g., cerebellar GM volume vs. whole cerebellum volume) [28,94]. Importantly, in our study, lower CWMF in PwPPMS was detected only at baseline. The possible explanation is that during observation, CWMF was stable in PwPPMS but in PwSPMS, it decreased, and the difference reached a non-statistical level.

Evaluating the dynamics of segmental volumes of different brain regions across the assessed MS subtypes, we showed that only in PwSPMS, RPF was significantly lower at follow-up than at baseline. No significant atrophy of the other assessed brain structures was detected during the observation, either in the whole study group or in its subgroups. Putamen, composed primarily of GM, is a part of the dorsal corpus striatum and is responsible for conscious movement and executive functions [95]. Its atrophy is common in many neurodegenerative diseases including MS [96]. Supporting our outcomes, an analysis of data from eight randomized, double-blind trials of a total of 1123 PwPPMS and 1282 PwSPMS showed more pronounced loss of GM and faster GM volume changes (including putamen) in PwSPMS than in PwPPMS [97]. In a very interesting study, Krämer et al. reported that the atrophy of putamen is present from the onset of RRMS and progresses degressively [98]. Importantly, two studies by the MAGNIMS group presented not fully consistent findings concerning the atrophy of putamen [16,27]. The first one [27] showed more pronounced putaminal atrophy in relapse-onset MS in comparison to PwPPMS, interpreted as a result of a greater inflammatory impact on this structure in PwRRMS. The other one [16] reported the fastest rate of putamen atrophy in PwPPMS. The possible association of the reduction of RPF in PwSPMS with longer disease duration than in PwPPMS in our study was not confirmed by the regression analysis. However, we believe that a longer observation period could potentially allow for better assessment and detection of atrophy in a group of PwPPMS [99].

Interestingly, we did not reveal significant differences in the rate of change of the investigated MRI parameters between the PMS subgroups throughout the observation period. This stays in line with the outcomes showing that the rates of the annualized brain volume change were not statistically different between PwPPMS and PwSPMS [100]. Our study may also support the recently published data indicating that in certain cases, disease duration has a lesser impact on clinical and radiological activity than age itself [101]. A similar rate of changes of the measured parameters might suggest that difference in disease duration between groups did not contribute to the cognitive and radiological progression as significantly as the participants’ age [101]. Additionally, no link between disease duration and thalamic and whole brain atrophy was reported in one study [102]. Another research project reported no correlation between disease duration and brain parenchymal fraction change, and only a weak correlation between disease duration and ventricular fraction change [103].

### 3.2. Cognitive Impairment in PwPPMS and PwSPMS

In the previous studies, the prevalence of CI was reported as 53% to 73% of PwPPMS and 55% to 86% of PwSPMS [104,105]. CI was found to occur more frequently and more severely in PwPMS than PwRRMS [104]. In PwSPMS, it is considered to be due to a longer disease duration and a higher lesion load [37], whereas in PwPPMS it might be partially explained by the more pronounced involvement of the brain cortex; for instance, a higher number of cortical lesions [19]. PwPMS are characterized also by a significantly higher level of cortical atrophy than PwRRMS, which is directly associated with cognition [44]. However, it remains inconclusive whether PwPPMS and PwSPMS have a similar pattern and prevalence of CI [104,105,106,107]. Some authors propose specific cognitive phenotypes in PwPPMS, consisting of the impairment of verbal learning and memory deficits [108] or the decline in visuospatial and language skills [109], while others deny such findings [110].

In the current study, as in our previous work [56], the cross-sectional analysis of the study population revealed that PwPPMS and PwSPMS had similar cognitive performances both at baseline and follow-up time points. These results are in accordance with some of the previous reports indicating that these two subtypes of MS follow a comparable CI, regarding severity, incidence and influenced domains [104,107,111,112,113].

During the observation period, a significant worsening of non-verbal memory, as assessed with BVMT-R, was observed in the whole study group, with a particularly pronounced effect in PwPPMS. Such a cognitive decline detected in PwPPMS (and not in PwSPMS) stays in concordance with lower CWMF in this subgroup. This cerebellar pathology was linked to the impaired visuospatial abilities, presenting as non-verbal memory deficits [114]. Also, a significant worsening of the overall BICAMS score in the PPMS subgroup was seen in the current analysis, as in our previous study on a comparable cohort of PwPMS [56]. No other significant alterations in cognitive functions of the study participants were found, which may be attributed to a relatively short period of observation.

### 3.3. Correlations Between MRI-Based Brain Atrophy and Cognitive Impairment in PwPPMS and PwSPMS

There is a fast-growing amount of data on the role of MRI in the evaluation of CI in MS, as it can reveal structural brain pathology underlying the cognitive deficits [48]. Studies showed that the atrophy of GM is the key player responsible for CI in various domains across all MS subtypes [44,45,46]. Noteworthy is that, in PwMS, GM atrophy seems to be more marked than WM atrophy [99] and importantly, it is GM pathology that contributes predominantly to the CI [115]. Nevertheless, the atrophy of WM is also important in the course of CI development and progression, as was demonstrated both in PwSPMS [43] and PwRRMS [116]. In a study based on diffusion tensor imaging (DTI), damage of WM was associated with CI in a mixed group of PwSPMS and PwRRMS [117] as well as in PwPPMS [118]. Mistri et al. found significant correlations of CI with both GM and WM structures—normalized GMV and fractional anisotropy (FA) of the medial lemniscus in PwPPMS—as well as with normalized WMV and FA of the fornix in PwSPMS [111]. An association between cerebellar atrophy and cognition was reported earlier on a cohort of 331 PwMS including 19 PwPPMS and 54 PwSPMS [119].

Our study revealed a set of significant correlations between several MRI-based brain atrophy and cognition parameters. Interestingly, both in the whole PMS group and in PwPPMS, worsening on SDMT was associated with the decrease in the same volumetric parameters (WM, right thalamus and total cerebellum). The exceptions were two correlations: between SDMT and both GMF and CWMF, present only in PwPMS as a whole, and the correlation between SDMT and CGMF in PwPPMS. Our results stay in line with the previous research demonstrating similar associations of brain atrophy and CI in PwPPMS and PwSPMS [70,120,121]. A recent meta-analysis by Mirmosayyeb et al., encompassing 136 studies and focused on relationships between various MRI and cognitive measurements in PwMS (N = 13822; without distinguishing between MS subtypes), showed that among the investigated parameters, thalamic volume had the most significant correlations with all three BICAMS subtests, including SDMT [120]. The correlation between thalamus atrophy and SDMT was also reported in another study on PwPMS [70] and PwPPMS [121]. Moreover, GM atrophy has been correlated with the occurrence and severity of cognitive deficits in both PwPPMS and PwSPMS [44]. In correspondence to our finding of the association between SDMT and cerebellar atrophy, in another study including 11 PwPPMS and 35 PwSPMS, the total volume of cerebellum explained some of the variance in SDMT [122]. A correlation of cerebellar pathology and IPS (assessed with SDMT) was also reported in a recent study in i.a. PwPMS [119]. Specifically in PwPPMS, association of GM atrophy of the left inferior semilunar lobe of cerebellum with SDMT was shown [123]. Interestingly, SDMT was also suggested as a predictor of the atrophy of specific cerebellum structures [94].

Conversely to PwPPMS results, in our group of PwSPMS only the results of the two Stroop subtests (SCWT-A and SCWT-B) correlated with atrophy parameters (GMF and CGMF, respectively). The association of local GM atrophy (thalamus and putamen) with an impaired IPS was reported also in another cohort including PwSPMS [124]. Decrease in thalamic volume was suggested as a correlate of worsened IPS also in another study based mainly on PwSPMS [125]. Although in both aforementioned analyses SDMT was used as a tool for IPS assessment, we believe that these findings stay in line with our observations. Also, the previously mentioned meta-analysis [120] presented a positive correlation between thalamic volume and the score of Delis–Kaplan Executive Function System [126], which is a composite scale assessing executive functions and including a modified SCWT. Furthermore, in the current study, the atrophy of cerebellar GM was associated with the deterioration of executive functions assessed with SCWT-B in PwSPMS, which is in accordance with the current understanding of the important contribution of the cerebellum to executive functions [127], and is supported by earlier observations in PwMS [119]. A meta-analysis by Ahmadian et al. concerning patients with isolated cerebellar damage reported a notably worse performance on both SCWT subtests (ergo worse IPS and executive functions) [114].

Despite a complex and multifactorial structure of the study, we found only a few differences in brain atrophy patterns between PwPPMS and PwSPMS. Most of the assessed brain volumetric parameters were comparable between these two subtypes of the disease. Additionally, the cognitive profile in both PMS subgroups was similar.

Thus, our results support the paradigm of MS as a continuum of clinical phenotypes with similar etiopathology driven by inflammation and neurodegeneration occurring simultaneously, both starting from an early beginning of disease and underlying clinical progression [128,129,130,131,132]. A growing body of evidence for this new model of MS comes from MRI and pathological studies reporting similar levels of cortical demyelination, axonal loss and inflammation in PwPPMS and PwSPMS [133,134,135,136]. However, demographic (age, sex) and genetic factors may have an impact on the diversity of clinical presentations of MS [137,138].

### 3.4. Potential Clinical Implications

The findings presented in this research, including only a few differences in MRI-based brain atrophy and cognitive assessment between PwPPMS and PwSPMS, add to the knowledge about the biology of progressive clinical subforms of MS, which has to be further developed in order to optimize the diagnostic criteria and treatment strategies in PwPMS. Additionally, correlations between some MRI data and SDMT underscore the value of this test in clinical practice as a gold standard for the assessment of cognitive functions in PwMS.

### 3.5. Future Directions

Further study including larger numbers of PwPMS, in comparison to RRMS, is planned in order to assess a broad spectrum of MS pathology in more detail. Additionally, future investigation combining MRI data, results from neuropsychological tests and serum biomarkers has to be considered as a more comprehensive analysis searching for markers of disease progression. Furthermore, to improve the physical disability assessment, a timed 25-foot walk test and 9-hole peg test should be incorporated. Also, it would be beneficial to include genetic factors such as, e.g., apolipoprotein E genotypes in the future analysis. Such investigations could possibly enable attempts to create statistical models for the prediction of CI in MS implementing deep learning algorithms, as was recently presented by Taloni et al. in the case of disability progression [139].

### 3.6. Study Limitations

The main limitation of this research is the relatively modest study group size. Although this study was designed as a prospective and very complex analysis performed in the real-world conditions, the results should be interpreted with caution. An approximately one-year follow-up should be considered as a preliminary observation; longer observation in larger population is intended based on the results of current study. Another limitation was the incorporation of two MRI scanners with different field strengths. Not accounting for the patients’ genetic profiles should be considered another limitation.

## 4. Materials and Methods

### 4.1. Study Group

The participants of this prospective, real-world, longitudinal study were recruited among the patients of the Department of Neurology and Neurological Outpatient Clinic at University Teaching Hospital No. 1 in Lodz, Poland. The study procedures were approved by the Bioethics Committee of Medical University of Lodz (decision Nos. RNN/128/20/KE and KE/564/23; date of approval: 12 May 2020). Authors performed this research in accordance with the Declaration of Helsinki (1964) and its latest revision.

The patients were included on the basis of written informed consent to participate in the study, age ≥18 and ≤70 years, and the diagnosis of PPMS or SPMS according to the diagnostic criteria [140]. SPMS diagnosis was grounded on the definition proposed by Lorscheider et al. [141]. The exclusion criteria included withdrawal of consent, diagnosis of RRMS, other concomitant CNS diseases or contraindication to MRI. Participants with a history of drug or alcohol abuse and those with a Beck Depression Inventory-II (BDI-II) [142] score over 19 points were also excluded from the study. During the study, three disease exacerbations were noted, and patients were treated with intravenous steroids. Consequently, in each instance, study assessments were conducted no earlier than three months following the relapse and the completion of steroidotherapy.

### 4.2. Study Procedures

#### 4.2.1. CI Assessment

The examination of CI encompassed several neuropsychological tests, including a screening battery for the assessment of CI in MS—Polish validation of BICAMS [143]. BICAMS comprises the Symbol Digit Modalities Test (SDMT) [144]—a test for IPS, as well as the first five recall trials of the California Verbal Learning Test-II (CVLT) and the first three recall trials of the Brief Visuospatial Memory Test Revised (BVMT-R)—tools for the assessment of verbal and non-verbal memory, respectively [145]. Patients with BICAMS scores of 1, 2 and 3 points were assigned to the cognitively impaired group, while those with 0 points were deemed free of a significant CI [143,146].

Moreover, the Stroop Color and Word Test (SCWT) was performed to complement BICAMS battery. SCWT is divided into two subtests: (1) SCWT-A (which involves reading color names written in black ink) measuring the work pace (and effectively—IPS) and (2) SCWT-B (which requires naming the color of the ink while inhibiting the automatic response to read the printed word, which itself is a color name), thereby serving as a measure of executive functions, particularly inhibitory control. Performance on this test is assessed by recording the time (in seconds) required to complete each task—higher times correspond to lower performance [147,148]. This scale is not validated in the Polish population, therefore no cut-off values are available.

#### 4.2.2. MRI Examination and Assessment

MRI examinations were performed on a 1.5 Tesla (T) scanner (Avanto Fit, Siemens Healthineers, Munich, Germany) or 3.0 T scanner (Magnetom Vida, Siemens Healthineers, Munich, Germany) with similar acquisition protocols, including the following sequences: 3-dimensional isometric T1-weighted axial sequence (3DT1), fluid attenuated inversion recovery (FLAIR) and 3DT1 after gadolinium contrast administration (0.1 mmol/kg BW). On 1.5T scanner, the parameters were as follows: 3DT1 magnetization prepared rapid gradient echo (MPRAGE): echo time (TE) 2.99 ms, repetition time (TR) 1610 ms, inversion time (TI) 1100 ms, slice thickness 0.9 mm, no gap, field of view (FOV) 256 × 256 mm; 2DFLAIR: TE 107 ms, TR 9000 ms, TI 2500 ms, slice thickness 3.0 mm, no gap, FOV 255 × 256 mm. On the 3T scanner, these parameters had the following values: 3DT1: TE 2.26 ms, TR 1800 ms, TI 900 ms, slice thickness 0.8 mm, no gap, FOV 256 × 256 mm; 3DFLAIR: TE 388 ms, TR 5000 ms, TI 1800 ms, slice thickness 0.8 mm, no gap, FOV 256 × 256mm. The segmentation of the brain structures was performed on 3DT1 in the automatic Exhibeon version 3 software (Pixel Technology LLC, Lodz, Poland; https://www.allerad.com/en/dicom-viewer (accessed on 7 July 2025)); for exemplary MRI images, see Figure S1. The segmentation procedure was described thoroughly in previous articles by Rościszewska-Żukowska et al. [149] and Puzio et al. [150].

The segmented structures were measured in milliliters (ml) and included the following: intracranial volume (ICV) [151], white matter volume (WMV), gray matter volume (GMV), brain cortical volume (BCV), left and right thalamic volume (LTV and RTV, respectively), left and right putaminal volume (LPV and RPV, respectively), left and right hippocampal volume (LHV and RHV, respectively), left and right choroid plexus volume (LCPV and RCPV, respectively), corpus callosum volume (CCV), cerebellar white matter volume (CWMV), cerebellar gray matter volume (CGMV) and total cerebellar volume (CV). The MRI segmentation was manually corrected by one of the authors with more than 25 years of experience in MRI analysis.

All MRI parameters were normalized to ICV (by dividing each measured volume by ICV) in order to minimalize intersubject variations in head size and subtle acquisition differences [151], and multiplied by 100 for clarity. Thereby, we obtained the normalized values, later referred to as fractions, expressed in percentage of ICV (%) and named as follows: white matter fraction (WMF), gray matter fraction (GMF), brain cortical fraction (BCF), left and right thalamic fraction (LTF and RTF, respectively), left and right putaminal fraction (LPF and RPF, respectively), left and right hippocampal fraction (LHF and RHF, respectively), left and right choroid plexus fraction (LCPF and RCPF, respectively), corpus callosum fraction (CCF), cerebellar white matter fraction (CWMF), cerebellar gray matter fraction (CGMF) and total cerebellar fraction (CF).

#### 4.2.3. Physical Disability Assessment

Physical disability was assessed with Expanded Disability Status Scale (EDSS) [152] and its progression was defined as an increase in EDSS by ≥1.0 points if baseline EDSS was ≤5.5 points and by ≥0.5 point if baseline EDSS was >5.5 points, according to Kappos et al. [153].

#### 4.2.4. Statistical Analysis

Statistical analyses were performed using Statistica 13 software (StatSoft, Tulsa, OK, USA) and GraphPad Prism 10 (GraphPad Software, Boston, MA, USA). Continuous variables are presented as means with standard deviations (SD), while categorical variables are presented as absolute numbers (N) and percentages. For non-normally distributed continuous variables, medians with interquartile ranges (IQR) were provided where applicable. The distribution of variables was assessed using the Shapiro–Wilk test. To compare differences between two independent groups, the Student’s *t*-test with Welch’s correction or Mann–Whitney U were applied. For paired comparisons between two time points within the same group, paired Student’s *t*-test or Wilcoxon signed-rank test were used. Correlations between continuous variables were assessed using the Spearman correlation coefficient. The strength of correlation was interpreted as follows: 0.00–0.19 very weak, 0.20–0.39 weak, 0.40–0.59 moderate, 0.60–0.79 strong, and 0.80–1.00 very strong. To evaluate the changes (Δ) in neuropsychological and neuroradiological parameters during the observation period, the mean differences were calculated and compared between subgroups. All tests were two-tailed, and a *p*-value below 0.05 was considered statistically significant. To evaluate the potential confounding effect of disease duration, we performed univariate linear regression analyses with each neuroimaging outcome as the dependent variable and disease duration as the independent variable.

## 5. Conclusions

The presented results indicate a very complex nature of progression in MS, demanding multifactorial and longitudinal assessment including, i.e., clinical and radiological factors. Importantly, in this one year-long prospective study with an assessment of a large number of volumetric MRI and cognitive parameters, only a few differences in MRI measures between PPMS and SPMS were observed, while most of the parameters were comparable between the PMS subgroups. These findings support similar characteristics of progressive pathological processes in people with various clinical presentations of progression in MS.

## Figures and Tables

**Table 1:** The baseline demographic and clinical characteristics of the study group. PwPMS—people with progressive multiple sclerosis, PwPPMS—people with primary progressive multiple sclerosis, PwSPMS—people with secondary progressive multiple sclerosis, F—female, M—male, EDSS—Expanded Disability Status Scale, HET—high-efficacy therapy, MET—moderate-efficacy therapy, DMT—disease modifying therapy. *p*-value < 0.05 written in bold. * *p*-value refers to differences in parameters between PwPPMS and PwSPMS.

Parameter		PwPMS (N = 39)		PwPPMS (N = 20)		PwSPMS (N = 19)		*p*-Value *
		Mean	SD	Mean	SD	Mean	SD	
Age [years]		54.51	7.77	54.40	7.07	54.63	8.64	0.9277
Sex [F/M]		27/12		13/7		14/5		0.8101
Years of education [years]		12.72	3.11	12.85	3.34	12.58	2.93	0.7890
Disease duration [years]		13.87	9.38	8.00	6.08	20.05	8.26	**0.00001**
EDSS baseline		5.18	1.14	4.75	1.16	5.63	0.96	**0.0137**
EDSS follow-up		5.51	1.16	5.13	1.28	5.92	0.87	**0.0287**
Therapy [N (%)]	HET	14 (36%)		10 (50%)		4 (21%)		
	MET	12 (31%)		0 (0%)		12 (63%)		
	other	2 (5%)		2 (10%)		0 (0%)		
	no DMT	11 (28%)		8 (40%)		3 (16%)		

**Table 2:** The changes (Δ) of the MRI fractions during the observation. PwPMS—people with progressive multiple sclerosis, PwPPMS—people with primary progressive multiple sclerosis, PwSPMS—people with secondary progressive multiple sclerosis, WMF—white matter fraction, GMF—gray matter fraction, BCF—brain cortical fraction, LTF—left thalamic fraction, RTF—right thalamic fraction, LPF—left putaminal fraction, RPF—right putaminal fraction, LHF—left hippocampal fraction, RHF—right hippocampal fraction, LCPF—left choroid plexus fraction, RCPF—right choroid plexus fraction, CCF—corpus callosum fraction, CWMF—cerebellar white matter fraction, CGMF—cerebellar gray matter fraction, CF—total cerebellar fraction. * *p*-value refers to differences in parameters between PwPPMS and PwSPMS.

Δ Fraction [%]	PwPMS (N = 39)		PwPPMS (N = 20)		PwSPMS (N = 19)		*p*-Value *
	Mean	SD	Mean	SD	Mean	SD	
ΔWMF	0.1081	2.8856	0.4147	3.9935	−0.2845	1.2447	0.4732
ΔGMF	−0.3491	2.9575	−0.3633	3.5637	−0.3168	2.3348	0.9621
ΔBCF	−0.2378	1.8664	−0.3410	2.6084	−0.1245	0.6986	0.7299
ΔLTF	0.0013	0.0330	−0.0001	0.0471	0.0024	0.0084	0.8263
ΔRTF	0.0021	0.0493	0.0034	0.0708	0.0004	0.0116	0.8553
ΔLPF	−0.0008	0.0214	−0.0006	0.0269	−0.0010	0.0152	0.9539
ΔRPF	−0.0041	0.0169	−0.0045	0.0227	−0.0045	0.0095	0.9945
ΔLHF	−0.0020	0.0237	−0.0013	0.0289	−0.0036	0.0178	0.7672
ΔRHF	−0.0016	0.0216	−0.0016	0.0294	−0.0019	0.0106	0.9643
ΔLCPF	−0.0001	0.0151	−0.0014	0.0197	0.0008	0.0094	0.6561
ΔRCPF	−0.0010	0.0178	−0.0024	0.0226	0.0006	0.0122	0.6130
ΔCCF	−0.0041	0.0215	−0.0026	0.0263	−0.0081	0.0191	0.4627
ΔCWMF	−0.0018	0.2313	0.0128	0.2663	−0.0418	0.2186	0.4893
ΔCGMF	0.0239	0.6762	0.0491	0.9634	0.0074	0.1804	0.8547
ΔCF	0.0221	0.8141	0.0620	1.1563	−0.0344	0.2558	0.7264

**Table 3:** The scores of the neuropsychological tests with regard to the study time point. PwPMS—people with progressive multiple sclerosis, PwPPMS—people with primary progressive multiple sclerosis, PwSPMS—people with secondary progressive multiple sclerosis, BICAMS—Brief International Cognitive Assessment for Multiple Sclerosis, SDMT—Symbol Digit Modalities Test, CVLT—California Verbal Learning Test, BVMT-R—Brief Visuospatial Memory Test Revised, SCWT-A—Stroop Color and Word Test subtest A, SCWT-B—Stroop Color and Word Test subtest B. *p*-value < 0.05 written in bold. * *p*-value refers to differences in parameters between PwPPMS and PwSPMS. ** *p*-value refers to differences in parameters between baseline and follow-up in each subgroup.

Test	Baseline						*p*-Value *	Follow-Up						*p*-Value *	*p*-Value **		
	PwPMS (N = 39)		PwPPMS (N = 20)		PwSPMS (N = 19)			PwPMS (N = 39)		PwPPMS (N = 20)		PwSPMS (N = 19)			PwPMS (N = 39)	PwPPMS (N = 20)	PwSPMS (N = 19)
	Mean	SD	Mean	SD	Mean	SD		Mean	SD	Mean	SD	Mean	SD				
BICAMS	1.21	0.86	1.05	0.89	1.37	0.83	0.2544	1.34	0.97	1.25	1.02	1.44	0.92	0.5408	0.0960	**0.0421**	0.6676
SDMT	32.51	11.24	32.95	13.96	32.05	7.79	0.8047	32.39	13.45	32.85	15.43	31.89	11.26	0.8266	0.9626	0.9486	1.0000
CVLT	50.00	10.73	51.50	8.99	48.42	12.35	0.3821	49.21	11.26	51.60	10.85	46.56	11.42	0.1725	0.8291	0.9371	0.7218
BVMT-R	18.74	7.43	19.50	8.29	17.89	6.48	0.5066	17.03	7.61	17.20	7.72	16.82	7.72	0.8833	**0.0209**	**0.0338**	0.3614
SCWT-A	28.97	7.07	28.90	9.03	29.05	4.43	0.9466	29.55	7.27	29.70	9.17	29.39	4.58	0.8941	0.4363	0.4183	0.8424
SCWT-B	71.74	24.25	75.16	28.64	68.32	19.08	0.3927	71.89	23.87	73.16	28.94	70.56	17.78	0.7426	0.9027	0.3193	0.3079

**Table 4:** The changes (Δ) of the neuropsychological tests during the observation. PwPMS—people with progressive multiple sclerosis, PwPPMS—people with primary progressive multiple sclerosis, PwSPMS—people with secondary progressive multiple sclerosis, BICAMS—Brief International Cognitive Assessment for Multiple Sclerosis, SDMT—Symbol Digit Modalities Test, CVLT—California Verbal Learning Test, BVMT-R—Brief Visuospatial Memory Test Revised, SCWT-A—Stroop Color and Word Test subtest A, SCWT-B—Stroop Color and Word Test subtest B. * *p*-value refers to differences in parameters between PwPPMS and PwSPMS.

Δ Test	PwPMS (N = 39)		PwPPMS (N = 20)		PwSPMS (N = 19)		*p*-Value *
	Mean	SD	Mean	SD	Mean	SD	
ΔBICAMS	0.10	0.50	0.20	0.41	0.00	0.58	0.2235
ΔSDMT	−0.95	8.79	−0.10	6.84	−1.84	10.58	0.5482
ΔCVLT	−2.05	12.92	0.10	5.59	−4.32	17.57	0.3070
ΔBVMT-R	−2.10	5.27	−2.30	4.50	−1.89	6.10	0.8155
ΔSCWT-A	−0.18	5.74	0.80	4.32	−1.21	6.90	0.2871
ΔSCWT-B	−0.16	8.01	−2.00	8.51	1.78	7.17	0.1527

## Data Availability

The original contributions presented in the study are included in the article/Supplementary Materials. Further inquiries can be directed to the corresponding author.

## Supplementary Materials

The following supporting information can be downloaded at https://www.mdpi.com/article/10.3390/ijms26178523/s1.

## Abbreviations

The following abbreviations are used in this manuscript:

MS	Multiple sclerosis
CNS	Central nervous system
PwMS	People with multiple sclerosis
RRMS	Relapsing-remitting multiple sclerosis
SPMS	Secondary progressive multiple sclerosis
PPMS	Primary progressive multiple sclerosis
PwPPMS	People with primary progressive multiple sclerosis
PwSPMS	People with secondary progressive multiple sclerosis
PwPMS	People with progressive multiple sclerosis
PMS	Progressive multiple sclerosis
MRI	Magnetic resonance imaging
GM	Gray matter
VBM	Voxel-based morphometry
CI	Cognitive impairment
IPS	Information processing speed
PwRRMS	People with relapsing-remitting multiple sclerosis
BICAMS	Brief International Cognitive Assessment in Multiple Sclerosis
BRB-N	Brief Repeatable Battery of Neuropsychological Tests
MACFIMS	Minimal Assessment of Cognitive Function in Multiple Sclerosis
WM	White matter
DMT	Disease modifying treatment
EDSS	Expanded Disability Status Scale
MET	Moderate-efficacy therapy
HET	High-efficacy therapy
F	Female
M	Male
LTF	Left thalamic fraction
CCF	Corpus callosum fraction
CWMF	Cerebellar white matter fraction
RPF	Right putaminal fraction
WMF	White matter fraction
GMF	Gray matter fraction
BCF	Brain cortical fraction
RTF	Right thalamic fraction
LPF	Left putaminal fraction
LHF	Left hippocampal fraction
RHF	Right hippocampal fraction
LCPF	Left choroid plexus fraction
RCPF	Right choroid plexus fraction
CGMF	Cerebellar gray matter fraction
CF	Total cerebellar fraction
BVMT-R	Brief Visuospatial Memory Test revised
SDMT	Symbol Digit Modalities Test
CVLT	California Learning Verbal Test
SCWT-A	Stroop Color and Word Test subtest A
SCWT-B	Stroop Color and Word Test subtest B
RIS	Radiologically isolated syndrome
CSF	Cerebrospinal fluid
CC	Corpus callosum
CIS	Clinically isolated syndrome
FA	Fractional anisotropy
DTI	Diffusion tensor imaging
BDI-II	Beck Depression Inventory-II
3DT1	3-dimensional isometric T1-weighted sequence
MPRAGE	Magnetization prepared rapid gradient echo
FLAIR	Fluid attenuated inversion recovery
BW	Body weight
TE	Echo time
TR	Repetition time
TI	Inversion time
FA	Flip angle
FOV	Field of view
ICV	Intracranial volume
WMV	White matter volume
GMV	Gray matter volume
BCV	Brain cortical volume
LTV	Left thalamic volume
RTV	Right thalamic volume
LPV	Left putaminal volume
RPV	Right putaminal volume
LHV	Left hippocampal volume
RHV	Right hippocampal volume
LCPV	Left choroid plexus volume
RCPV	Right choroid plexus volume
CCV	Corpus callosum volume
CWMV	Cerebellar white matter volume
CGMV	Cerebellar gray matter volume
CV	Total cerebellar volume
