# Supplementary material for: Brain Atrophy and Cognitive Impairment in Primary and Secondary Progressive Multiple Sclerosis Cohort—Similar Progressive MS Phenotype
Source: Int J Mol Sci. 2025 Sep 2;26(17):8523. doi: 10.3390/ijms26178523 (PMC12429507; doi:10.3390/ijms26178523)
Supplement: Supplementary file 1 [file ijms-26-08523-s001.zip › ijms-3809928-supplementary.pdf]

Supplementary Materials

Table S1. The MRI fractions with regard to the study time point. MRI – magnetic resonance imaging, PwPMS – people with progressive multiple sclerosis, PwPPMS – people with primary progressive multiple sclerosis, PwSPMS – people with secondary progressive multiple sclerosis, WMF – white matter fraction, GMF – grey matter fraction, BCF – brain cortical fraction, LTF – left thalamic fraction, RTF – right thalamic fraction, LPF – left putaminal fraction, RPF – right putaminal fraction, LHF – left hippocampal fraction, RHF – right hippocampal fraction, LCPF – left choroid plexus fraction, RCPF – right choroid plexus fraction, CCF – corpus callosum fraction, CWMF – cerebellar white matter fraction, CGMF – cerebellar grey matter fraction, CF – total cerebellar fraction. P-value <0.05 written in bold. * P-value refers to differences in parameters between PwPPMS and PwSPMS. ** P-value refers to differences in parameters between baseline and follow-up in each subgroup.

MRI fractions [%]	baseline						p-value*	follow-up						p-value*	p-value**		
	PwPMS (N=39)		PwPPMS (N=20)		PwSPMS (N=19)			PwPMS (N=39)		PwPPMS (N=20)		PwSPMS (N=19)			PwPMS (N=39)	PwPPMS (N=20)	PwSPMS (N=19)
	Mean	SD	Mean	SD	Mean	SD		Mean	SD	Mean	SD	Mean	SD				
WMF	30.41	2.90	30.94	3.91	29.91	1.36	0.3518	30.47	3.23	31.36	4.18	29.62	1.66	0.1556	0.9046	0.6562	0.3196
GMF	36.17	3.37	37.15	3.84	35.24	2.64	0.0817	35.83	3.40	36.78	3.96	34.92	2.55	0.0967	0.4778	0.6621	0.5511
BCF	30.69	2.25	31.29	2.57	30.13	1.78	0.1241	30.46	2.27	30.95	2.71	30.00	1.69	0.2386	0.4462	0.5759	0.4355
LTF	0.42	0.06	0.44	0.06	0.39	0.04	**0.0203**	0.42	0.06	0.44	0.07	0.40	0.04	**0.0443**	0.8253	0.9943	0.2242
RTF	0.41	0.07	0.44	0.08	0.39	0.04	0.0500	0.42	0.08	0.44	0.10	0.39	0.04	0.0845	0.8169	0.8363	0.8901
LPF	0.35	0.04	0.36	0.03	0.35	0.04	0.6308	0.35	0.05	0.36	0.05	0.35	0.04	0.6810	0.8215	0.9276	0.7769
RPF	0.34	0.04	0.34	0.04	0.332	0.05	0.8191	0.33	0.05	0.34	0.04	0.328	0.05	0.8651	0.1053	0.3948	**0.0479**
LHF	0.30	0.03	0.31	0.04	0.30	0.03	0.8301	0.30	0.04	0.30	0.05	0.30	0.03	0.9457	0.5163	0.8483	0.3764
RHF	0.30	0.04	0.31	0.04	0.30	0.04	0.5860	0.30	0.04	0.31	0.05	0.30	0.04	0.5902	0.6181	0.8184	0.4342
LCPF	0.05	0.03	0.05	0.03	0.05	0.02	0.5000	0.05	0.02	0.05	0.02	0.05	0.02	0.6882	0.9105	0.7561	0.7004
RCPF	0.05	0.02	0.05	0.02	0.04	0.02	0.2273	0.05	0.02	0.05	0.02	0.04	0.02	0.2823	0.7700	0.6516	0.8236
CCF	0.28	0.05	0.30	0.05	0.26	0.05	**0.0097**	0.27	0.06	0.30	0.05	0.25	0.06	**0.0103**	0.1475	0.6751	0.0745
CWMF	1.92	0.23	1.83	0.20	2.01	0.24	**0.0132**	1.91	0.25	1.84	0.25	1.97	0.23	0.0543	0.6965	0.8358	0.4029
CGMF	6.57	0.91	6.64	1.13	6.50	0.66	0.7606	6.60	0.92	6.69	1.13	6.50	0.68	0.6864	0.7989	0.8266	0.8556
CF	8.49	1.05	8.47	1.30	8.51	0.77	0.7776	8.50	1.09	8.53	1.32	8.47	0.84	0.9354	0.9242	0.8180	0.5547

Table S2. Correlations between the changes (Δ) of MRI fractions and neuropsychological tests' scores. PwPMS (N=39) – people with progressive multiple sclerosis, PwPPMS (N=20) – people with primary progressive multiple sclerosis, PwSPMS (N=19) – people with secondary progressive multiple sclerosis, WMF – white matter fraction, GMF – grey matter fraction, BCF – brain cortical fraction, LTF – left thalamic fraction, RTF – right thalamic fraction, LPF – left putaminal fraction, RPF – right putaminal fraction, LHF – left hippocampal fraction, RHF – right hippocampal fraction, LCPF – left choroid plexus fraction, RCPF – right choroid plexus fraction, CCF – corpus callosum fraction, CWMF – cerebellar white matter fraction, CGMF – cerebellar grey matter fraction, CF – total cerebellar fraction, BICAMS – Brief International Cognitive Assessment for Multiple Sclerosis, SDMT – Symbol Digit Modalities Test, CVLT – California Verbal Learning Test, BVMT-R – Brief Visuospatial Memory Test Revised, SCWT-A – Stroop Color and Word Test subtest A, SCWT-B – Stroop Color and Word Test subtest B. P-value <0.05 written in bold. * P-value refers to the statistical significance of the correlations between the changes of the measured parameters.

		PwPMS	PwPPMS	PwSPMS	PwPMS	PwPPMS	PwSPMS	PwPMS	PwPPMS	PwSPMS	PwPMS	PwPPMS	PwSPMS	PwPMS	PwPPMS	PwSPMS	PwPMS	PwPPMS	PwSPMS	PwPMS	PwPPMS	PwSPMS
		ΔWMF			ΔGMF			ΔBCF			ΔLTF			ΔRTF			ΔLPF			ΔRPF		
ΔBICAMS	R	-0.08	-0.12	-0.13	-0.04	0.12	-0.27	-0.08	0.01	-0.41	0.00	0.08	-0.28	0.01	0.05	-0.23	0.00	0.06	-0.09	-0.04	0.07	-0.27
	p-value*	0.64	0.64	0.62	0.80	0.62	0.28	0.65	0.97	0.09	0.98	0.73	0.25	0.97	0.84	0.37	1.00	0.79	0.74	0.81	0.77	0.28
ΔSDMT	R	**0.43**	**0.54**	0.32	**0.34**	0.40	0.26	0.26	0.35	0.12	0.33	0.45	0.09	**0.37**	**0.52**	0.01	0.26	0.41	0.03	0.16	0.31	-0.18
	p-value*	**0.01**	**0.02**	0.19	**0.04**	0.09	0.30	0.12	0.14	0.65	0.05	0.05	0.74	**0.02**	**0.02**	0.98	0.11	0.09	0.89	0.36	0.20	0.48
ΔCVLT	R	-0.07	-0.08	-0.13	-0.06	-0.04	-0.12	0.06	0.02	0.31	-0.06	-0.12	0.11	-0.03	-0.09	0.22	-0.04	0.09	-0.24	0.01	0.01	0.02
	p-value*	0.70	0.75	0.61	0.72	0.86	0.64	0.70	0.93	0.21	0.71	0.63	0.66	0.86	0.72	0.39	0.82	0.71	0.33	0.95	0.98	0.94
ΔBVMT-R	R	-0.20	-0.21	-0.09	-0.20	-0.31	0.13	-0.15	-0.18	-0.14	-0.25	-0.31	-0.09	-0.28	-0.33	-0.01	-0.02	-0.18	0.44	-0.12	-0.23	0.30
	p-value*	0.23	0.39	0.72	0.23	0.20	0.62	0.37	0.46	0.58	0.14	0.20	0.73	0.10	0.16	0.97	0.92	0.47	0.07	0.49	0.35	0.22
ΔSCWT-A	R	-0.06	-0.03	-0.30	-0.15	0.02	**-0.52**	-0.10	-0.06	-0.33	-0.04	-0.02	-0.18	-0.05	-0.06	-0.15	-0.08	-0.13	0.03	-0.02	-0.02	-0.02
	p-value*	0.71	0.90	0.23	0.39	0.95	**0.03**	0.57	0.81	0.18	0.81	0.93	0.46	0.75	0.82	0.55	0.64	0.59	0.91	0.92	0.93	0.95
ΔSCWT-B	R	0.08	0.10	0.22	0.08	0.07	0.16	0.07	0.11	-0.13	0.12	0.20	-0.35	0.15	0.22	-0.09	0.23	0.21	0.33	0.09	0.06	0.22
	p-value*	0.62	0.69	0.38	0.65	0.78	0.53	0.67	0.65	0.62	0.49	0.42	0.15	0.38	0.37	0.73	0.17	0.39	0.18	0.61	0.82	0.38

Table S2. – Cont.

	PwPMS	PwPPMS	PwSPMS	PwPMS	PwPPMS	PwSPMS	PwPMS	PwPPMS	PwSPMS	PwPMS	PwPPMS	PwSPMS	PwPMS	PwPPMS	PwSPMS	PwPMS	PwPPMS	PwSPMS	PwPMS	PwPPMS	PwSPMS	PwPMS	PwPPMS	PwSPMS
	ΔLHF			ΔRHF			ΔLCPF			ΔRCPF			ΔCCF			ΔCWMF			ΔCGMF			ΔCF		
ΔBICAMS	-0.15	-0.07	-0.30	-0.07	0.01	-0.29	-0.04	-0.11	0.09	0.00	-0.06	0.10	-0.10	0.13	-0.40	-0.27	-0.34	-0.26	0.00	-0.04	0.14	-0.08	-0.12	-0.11
	0.37	0.79	0.22	0.66	0.98	0.25	0.84	0.67	0.72	1.00	0.81	0.68	0.57	0.60	0.10	0.10	0.15	0.30	1.00	0.86	0.59	0.65	0.64	0.65
ΔSDMT	0.26	0.36	0.10	0.25	0.41	-0.10	0.23	0.44	-0.17	0.14	0.34	-0.23	0.17	0.29	-0.01	**0.37**	0.41	0.31	0.31	**0.48**	-0.24	**0.36**	**0.50**	0.07
	0.13	0.13	0.69	0.13	0.08	0.70	0.17	0.06	0.50	0.42	0.15	0.36	0.31	0.24	0.98	**0.03**	0.08	0.21	0.07	**0.04**	0.33	**0.03**	**0.03**	0.77
ΔCVLT	0.05	-0.02	0.13	0.04	0.03	0.07	0.16	0.22	0.13	0.22	0.30	0.16	0.15	0.04	0.29	0.12	0.06	0.18	0.04	0.05	-0.01	0.06	0.06	0.15
	0.77	0.94	0.62	0.80	0.89	0.78	0.34	0.36	0.60	0.20	0.21	0.53	0.37	0.86	0.24	0.46	0.79	0.47	0.84	0.83	0.98	0.71	0.81	0.56
ΔBVMT-R	-0.16	-0.10	-0.26	-0.23	-0.28	-0.05	0.03	-0.02	0.11	-0.01	0.00	-0.11	-0.08	-0.18	0.23	-0.03	0.03	-0.08	-0.15	-0.18	0.01	-0.13	-0.15	-0.06
	0.33	0.67	0.29	0.17	0.25	0.85	0.84	0.94	0.67	0.97	0.99	0.66	0.62	0.46	0.35	0.88	0.91	0.75	0.37	0.45	0.97	0.43	0.55	0.82
ΔSCWT-A	0.01	0.08	-0.16	0.01	0.03	-0.06	0.01	-0.03	0.18	0.03	0.00	0.12	-0.02	0.10	-0.30	-0.14	-0.03	-0.35	-0.01	-0.03	0.12	-0.04	-0.03	-0.20
	0.94	0.73	0.53	0.94	0.91	0.82	0.94	0.89	0.47	0.86	0.99	0.62	0.90	0.68	0.22	0.42	0.91	0.16	0.98	0.91	0.62	0.80	0.90	0.44
ΔSCWT-B	0.12	0.18	0.06	0.07	0.09	0.09	0.12	0.16	-0.05	0.00	0.05	-0.18	0.05	0.11	0.01	0.10	0.13	0.10	0.02	0.12	**-0.51**	0.04	0.13	-0.30
	0.49	0.45	0.82	0.66	0.71	0.74	0.48	0.51	0.85	1.00	0.84	0.48	0.76	0.66	0.97	0.56	0.59	0.68	0.91	0.64	**0.03**	0.80	0.60	0.22

Table S3. Univariate linear regression analysis for disease duration and brain atrophy parameters (Δ). PwPMS – people with progressive multiple sclerosis, PwPPMS – people with primary progressive multiple sclerosis, PwSPMS – people with secondary progressive multiple sclerosis, WMF – white matter fraction, GMF – grey matter fraction, BCF – brain cortical fraction, LTF – left thalamic fraction, RTF – right thalamic fraction, LPF – left putaminal fraction, RPF – right putaminal fraction, LHF – left hippocampal fraction, RHF – right hippocampal fraction, LCPF – left choroid plexus fraction, RCPF – right choroid plexus fraction, CCF – corpus callosum fraction, CWMF – cerebellar white matter fraction, CGMF – cerebellar grey matter fraction, CF – total cerebellar fraction, Beta – regression coefficient. * P-value refers to the statistical significance of the model in each subgroup.

Δ Fraction [%]	PwPMS (N=39)		PwPPMS (N=20)		PwSPMS (N=19)	
	Beta	p-value*	Beta	p-value*	Beta	p-value*
ΔWMF	0.0153	0.93	0.0336	0.89	0.3837	0.11
ΔGMF	0.0335	0.84	-0.0169	0.94	0.0947	0.70
ΔBCF	0.0107	0.95	-0.1646	0.49	0.2493	0.30
ΔLTF	0.0506	0.76	0.0177	0.94	0.1034	0.67
ΔRTF	0.0337	0.84	0.0340	0.89	0.2888	0.23
ΔLPF	-0.0477	0.77	-0.0536	0.82	-0.0761	0.76
ΔRPF	0.0233	0.89	-0.0067	0.98	0.0622	0.80
ΔLHF	0.0764	0.64	-0.0236	0.92	0.3901	0.10
ΔRHF	-0.0114	0.95	0.0018	0.99	-0.0099	0.97
ΔLCPF	-0.1828	0.27	-0.3162	0.17	-0.4079	0.08
ΔRCPF	-0.1551	0.35	-0.3642	0.11	-0.1812	0.46
ΔCCF	-0.1631	0.32	-0.4423	0.05	0.1796	0.46
ΔCWMF	0.1202	0.47	0.0275	0.91	0.4458	0.06
ΔCGMF	-0.0188	0.91	0.0833	0.73	-0.3655	0.12
ΔCF	0.0185	0.91	0.0758	0.75	0.0935	0.70

Table S4. Univariate linear regression analysis for disease duration and brain atrophy parameters (fractions) at both study time points. PwPMS – people with progressive multiple sclerosis, PwPPMS – people with primary progressive multiple sclerosis, PwSPMS – people with secondary progressive multiple sclerosis, WMF – white matter fraction, GMF – grey matter fraction, BCF – brain cortical fraction, LTF – left thalamic fraction, RTF – right thalamic fraction, LPF – left putaminal fraction, RPF – right putaminal fraction, LHF – left hippocampal fraction, RHF – right hippocampal fraction, LCPF – left choroid plexus fraction, RCPF – right choroid plexus fraction, CCF – corpus callosum fraction, CWMF – cerebellar white matter fraction, CGMF – cerebellar grey matter fraction, CF – total cerebellar fraction, Beta – regression coefficient. P-value <0.05 written in bold. * P-value refers to the statistical significance of the model in each subgroup at each study time point.

MRI fractions [%]	baseline						follow-up					
	PwPMS (N=39)		PwPPMS (N=20)		PwSPMS (N=19)		PwPMS (N=39)		PwPPMS (N=20)		PwSPMS (N=19)	
	Beta	p-value*	Beta	p-value*	Beta	p-value*	Beta	p-value*	Beta	p-value*	Beta	p-value*
WMF	-0.0573	p=0.729	0.2905	p=0.214	-0.4248	p=0.070	-0.0384	p=0.816	0.3025	p=0.195	-0.0779	p=0.751
GMF	-0.2741	p=0.091	0.1492	p=0.530	-0.4404	p=0.059	-0.2429	p=0.136	0.1294	p=0.587	-0.3682	p=0.121
BCF	-0.2465	p=0.130	0.1491	p=0.530	-0.4258	p=0.069	-0.2366	p=0.147	-0.0169	p=0.944	-0.3483	p=0.144
LTF	-0.2317	p=0.156	0.2083	p=0.378	-0.2367	p=0.329	-0.1913	p=0.243	0.1969	p=0.405	-0.2352	p=0.332
RTF	-0.1325	p=0.421	0.2483	p=0.291	-0.1201	p=0.624	-0.0916	p=0.579	0.2310	p=0.327	-0.0294	p=0.905
LPF	-0.0998	p=0.546	0.2395	p=0.309	-0.2727	p=0.259	-0.1045	p=0.527	0.1412	p=0.553	-0.2907	p=0.227

RPF	-0.0349	p=0.833	0.3380	p=0.145	-0.2272	p=0.350	-0.0232	p=0.888	0.2720	p=0.246	-0.2205	p=0.364
LHF	0.1018	p=0.538	0.2751	p=0.240	-0.0885	p=0.719	0.1382	p=0.401	0.2071	p=0.381	0.2266	p=0.351
RHF	0.0555	p=0.737	0.1300	p=0.585	0.1929	p=0.429	0.0423	p=0.798	0.1140	p=0.632	0.1750	p=0.474
LCPF	0.0815	p=0.622	0.1352	p=0.570	0.3398	p=0.155	-0.0424	p=0.798	-0.0966	p=0.685	0.0974	p=0.692
RCPF	0.0360	p=0.828	0.2229	p=0.345	0.2590	p=0.284	-0.1240	p=0.452	-0.1356	p=0.569	0.1129	p=0.645
CCF	-0.3135	p=0.052	0.1272	p=0.593	-0.2183	p=0.369	**-0.3540**	**p=0.027**	-0.1177	p=0.621	-0.1371	p=0.576
CWMF	0.2569	p=0.114	0.1362	p=0.567	-0.0878	p=0.721	**0.3666**	**p=0.022**	0.1354	p=0.569	0.3243	p=0.176
CGMF	0.0297	p=0.858	0.1468	p=0.537	0.0128	p=0.958	0.0154	p=0.926	0.2176	p=0.357	-0.0883	p=0.719
CF	0.0836	p=0.613	0.1491	p=0.531	-0.0166	p=0.946	0.0950	p=0.565	0.2113	p=0.371	0.0120	p=0.961

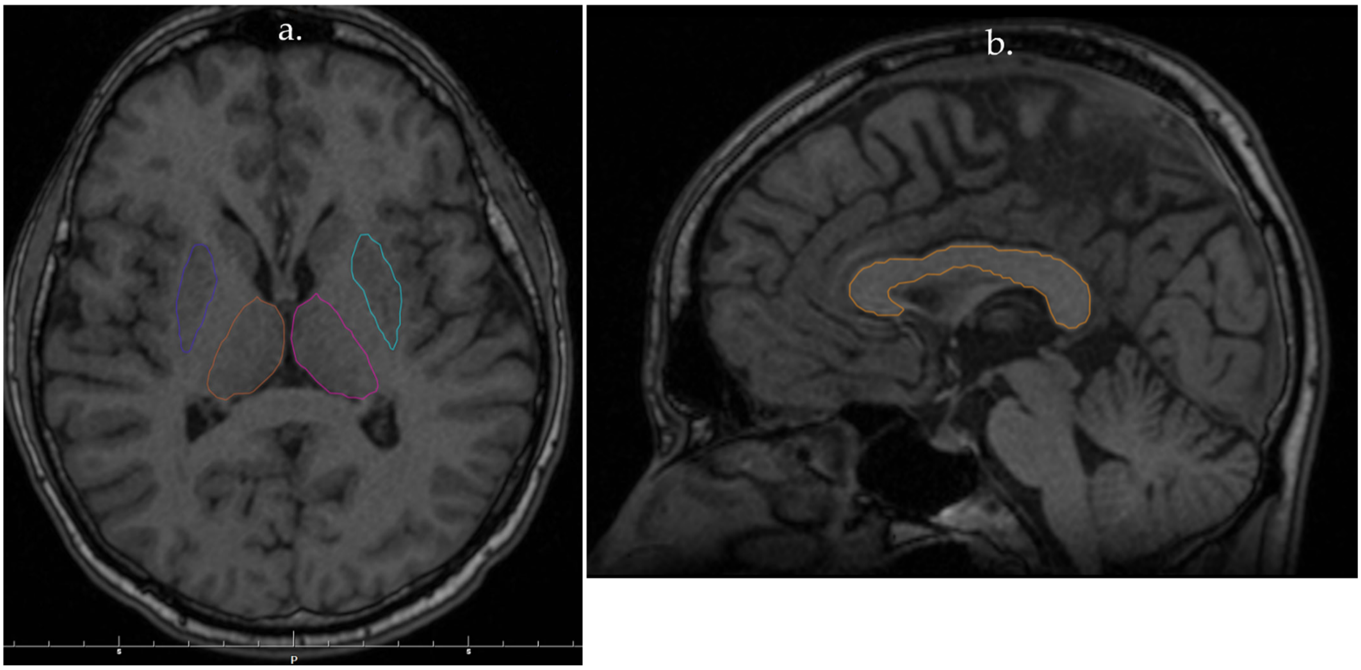

Figure S1. Examples of MRI segmentation. (a) 3DT1 axial image of left and right thalami and left and right putamina; (b) 3DT1 sagittal image of corpus callosum. MRI – magnetic resonance imaging, 3DT1 – 3-dimensional isometric T1-weighted sequence.
